# High-throughput cell-based assays for identifying antagonists of multiple smoking-associated human nicotinic acetylcholine receptor subtypes

**DOI:** 10.1016/j.slasd.2021.10.001

**Published:** 2021-10-09

**Authors:** Michelle Kassner, J. Brek Eaton, Nanyun Tang, Joachim L. Petit, Nathalie Meurice, Hongwei Holly Yin, Paul Whiteaker

**Affiliations:** a Cancer and Cell Biology Division, Translational Genomics Research Institute, Phoenix, AZ 85004, United States; b Division of Neurobiology, Barrow Neurological Institute, St. Joseph’s Hospital and Medical Center, 350 W. Thomas Rd., Phoenix, AZ 85013, United States; c Department of Hematology/Oncology, Mayo Clinic, Scottsdale, AZ 85259, United States

**Keywords:** Nicotinic acetylcholine receptor, Membrane potential assays, Cell-based screening

## Abstract

There is substantial evidence that in addition to nicotine, other compounds found in tobacco smoke significantly influence smoking behavior. Further, recent years have seen an explosion in the availability of non-combusted products that deliver nicotine, such as e-cigarettes and “home-brew” vaping devices that are essentially unregulated. There are many thousands of compounds in tobacco smoke alone, and new products are constantly introducing new compounds. Uncovering which of these compounds are active, across multiple smoking-relevant subtypes of the nicotinic acetylcholine receptor (nAChR) that influence tobacco/nicotine addiction, requires a high-throughput screening (HTS) approach. Accordingly, we developed a panel of HTS-friendly cell-based assays, all performed in the same cellular background and using the same membrane potential dye readout, to measure the function of the *α*3*β*4-, *α*4*β*2-, and *α*6*β*2-nAChR subtypes. These subtypes have each been prominently and consistently associated with human smoking behavior. We validated our assays by performing pilot screening of an expanded set of the Prestwick FDA-approved drug library. The screens displayed excellent performance parameters, and moderate hit rates (mean of 1.2% across all three assays) were achieved when identifying antagonists (chosen since effects of endogenous antagonists on consumption of nicotine/tobacco products are under-studied). Validation rates using an orthogonal assay (^86^Rb^+^ efflux) averaged 73% across the three assays. The resulting panel of assays represents a valuable new platform with which to screen and identify nAChR subtype-selective compounds. This provides a resource for identifying smoking-related compounds in both combusted and non-combusted tobacco products, with potential relevance in the search for additional smoking-cessation therapies.

## Introduction

Despite decades of public health awareness campaigns, smoking remains prevalent [1]. The health consequences of smoking are severe [2], and tobacco use is estimated to kill 50% of users [3], causing more deaths than all other abused substances combined.

Nicotine, the major alkaloid in tobacco, is present in cigarette smoke at ≈1 mg dose per cigarette and is the primary addictive component of tobacco products. Across all tobacco products, activity at neurotransmitter receptors called nicotinic acetylcholine receptors (nAChR) underlies the addictive nature of tobacco products [4]. In particular, a small number of heteromeric nAChR subtypes play predominant, well-defined roles in smoking behavior. *α*4*β*2*-nAChR (*indicates the possible presence of additional subunits [5]) is the most prevalent central nervous system (CNS) subtype [6], and human genome-wide association studies (GWAS) and clinical studies have indicated its relevance to tobacco use and nicotine dependence. Studies using animal models have confirmed roles for *α*4*β*2*-nAChR in tobacco use initiation and dependence [7, 8], likely mediated by enhanced *α*4*β*2*-nAChR function in the nigrostriatal reward pathway, a major dopamine pathway in the brain [9]. Although expression of *α*6*β*2*-nAChR is much more restricted, this subtype is also prevalent on mesolimbic dopamine projections, including the nigrostriatal reward pathway [10, 11]. GWAS studies have strongly associated human *α*6*-nAChR with smoking-related behaviors, and further associations have been found with the human CHRNB3 gene that encodes the *β*3-nAChR subunit; *β*3 are found within ≥75% of naturally expressed *α*6*-nAChR [12]. In addition, polymorphisms within the CHRNA5-CHRNA3-CHRNB4 gene cluster that encodes the nAChR *α*5, *α*3, and *β*4 subunits, respectively, are strongly associated with variations in tobacco use and dependence across multiple human populations [13]. Animal models investigating the *α*3*β*4*-nAChR subtype, especially those located in the habenulopeduncular tract within the midbrain, have shown a role in nicotine dependence, withdrawal, and aversive behavior [14, 15].

In addition to nicotine, tobacco smoke contains a complex mix of compounds that may also have relevance to smoking behavior. Perhaps the best-established evidence is for non-nicotine tobacco alkaloids (NNTAs), including anabasine, anatabine, cotinine, nornicotine, myosmine, and nicotyrine. When coupled with nicotine, NNTAs have reinforcing effects. For example, intravenous administration of a “cocktail” of nicotine with five other NNTAs (at the same ratios found in tobacco) elicited significantly greater increases in locomotor activity and self-administration behavior than did equivalent nicotine-only doses [16]. In a second study, combination of nicotine and NNTAs increased dopamine release in the nucleus accumbens reward center compared to an equivalent nicotine-only dose [17]. Research with individual NNTAs has also revealed pharmacologic actions that may contribute to tobacco dependence [18]. Further, there is a growing realization that additives such as menthol and green-apple flavor may also impact the function of nAChR subtypes implicated in tobacco addiction phenotypes [19, 20]. Importantly smokers, even after switching from their usual brands to very low nicotine content cigarettes, continue to smoke and this behavior can be sustained for up to six months [21]. This suggests that non-nicotine compounds significantly contribute to smoking behavior by modifying the effects of nicotine at nAChR subtypes and may even substitute for nicotine in low-nicotine cigarettes. Most studies to date have focused on the effects on smoking/consumption of agonists found within tobacco products (in addition to nicotine). However, additional antagonists (as considered in this study) could also influence consumption of products. For example, compounds that antagonize *α*4*β*2- and *α*6*β*2-nAChR function would be predicted to reduce the rewarding effects of nicotine consumption that are mediated by these two subtypes [7–12]. This would be expected to reduce the addictive potential of nicotine. Antagonists of *α*3*β*4-nAChR would be predicted to reduce the aversive effects of nicotine that limit consumption [14], an effect that might increase the dependence potential of nicotine. Since *α*3*β*4-nAChR also mediate aspects of nicotine withdrawal following abstinence from nicotine consumption [15], ingesting antagonists of *α*3*β*4-nAChR would be expected to alleviate withdrawal symptoms. This might make them useful therapeutics (by themselves), but could also contribute to the urge to reduce withdrawal by relapsing to consuming nicotine/tobacco products.

Since passage of the Family Smoking Prevention and Tobacco Control Act (FSPTCA) in 2009, the US Food and Drug Administration (FDA) has been responsible for regulating all tobacco products, both combusted and non-combusted, and both existing and novel products. A critical question arises: “what components of tobacco products should be regulated?” Although it is well understood that nicotine is the major psychoactive component of tobacco, other tobacco compounds may also have significant impacts, as described above. Because cigarette smoke contains many thousands of compounds [22], determining which are active—across multiple smoking-relevant nAChR subtypes—requires a high-throughput screening (HTS) approach. With the continuing appearance of novel non-combusted tobacco/nicotine products, the scale of this challenge will only increase. A shift to reliable HTS assays is thus an essential innovation for understanding the underlying pharmacology driving smoking behavior, since previouslyused lower-throughput methods simply do not have the capacity to identify efficiently compounds active at one or more of the multiple nAChR subtypes relevant to tobacco/nicotine use, within the large and expanding gamut of compounds found in such products. Once such compounds of interest are identified, they could be studied in detail using lower-throughput assays that could focus, for example, on how they (singly, or in combination) modify the action of nicotine at the levels of nAChR subtype function and/or animal models of behaviors related to nicotine/tobacco use disorder. Such studies would follow the examples of those cited in the preceding paragraph, but would expand significantly their scope. Further, the establishment of assays capable of superb high-throughput performance (i.e., robustness and reproducibility measured by Coefficient of Variation (CV) < 10%, Signal-to-Background (S:B) Ratio > 4, and Z’-factor > 0.5, https://www.ncbi.nlm.nih.gov/books/NBK53196/) will provide useful tools for drug discovery and development, notably including for smoking-cessation pharmacotherapies.

In this study, we describe the development, optimization, and validation of HTS-capable, cell-based assays for functional activity of the major heteromeric subtypes of nAChR (*α*3*β*4*, *α*4*β*2*, and *α*6*β*2*) with proven relevance to tobacco use initiation and dependence. We used the SH-EP1 human neuroblastoma cell line, which does not naturally express any nAChR subunit genes but has proved to be a receptive host [23], as a starting point. In this background, we stably expressed defined subtypes of interest, for the development and optimization of HTS to identify antagonists of nAChRs. We measured the changes in membrane potential associated with nAChR activity stimulated by nicotine, in the presence and absence of test compounds, allowing identification of antagonists. We further demonstrated the suitability of these assays for HTS by conducting a pilot compound screening of 2,298 compounds with automation, followed by validation of hits using an orthogonal assay. Our approach identified a set of nAChR compounds with confirmed nAChR antagonist behavior, several of which are subtype selective, and others that exhibit similar potency across multiple subtypes.

## Materials and methods

### Chemicals

All chemicals were purchased from Sigma unless otherwise specified.

### Construction and maintenance of stably-transfected α3β4, α4β2, and α6/3 β2 β3 cell lines

The engineering of the stably-transfected SH-EP1-*α*6/3 *β*2*β*3-nAChR cell line was initially described [24] for use in a voltage-sensitive FLIPR/Ca^2+^ dye functional assay. As described in that prior publication, this SH-EP1-*α*6/3 *β*2*β*3-nAChR monoclone is maintained in medium supplemented with zeocin (0.25 mg/mL), hygromycin (0.13 mg/mL biologically active) and G418 (0.6 mg/mL) to maintain positive selection of transfectants, and cultured at low passage numbers (1–40 from our frozen stocks) to ensure stable expression of the phenotype. Two new SH-EP1-*α*3*β*4-nAChR and SH-EP1-*α*4*β*2-nAChR lines were created and maintained in essentially the same way as described in [24]. In each case, pcDNA3.1zeo was used to introduce the respective *α* subunits and pcDNA3.1hygro for the respective *β* subunits. Human nAChR subunit genes optimized for vertebrate expression were used, which were designed to produce amino-acid sequences corresponding to human consensus sequences of the *α*3, *α*4, *β*2, or *β*4 subunits (synthesized by Invitrogen GeneArt, ThermoFisher Scientific). Importantly, artificially expressed nAChR subtypes have been shown consistently to reproduce faithfully the functional pharmacology of their naturally-expressed counterparts (including those subtypes studied here [24–26]).

All three cell lines were typically passaged once weekly by splitting cultures 1/20–1/40, just before reaching confluency, to maintain cells in a proliferative growth phase.

### Compound library plate preparation

The Prestwick chemical library (PCL ver.10) was purchased from Prestwick Chemicals; it consists of 1200 approved, off-patent drugs with high chemical and pharmacological diversity. An additional 1098 structurally diverse discovery compounds from the Mayo Compound Collection were added to the pilot screen deck. These compounds were selected from the ChemDiv CNS-targeted library. Compounds at 10mM concentration (100% DMSO) were arrayed into 384-well source plates using a Biomek NXP Span8 (Beckman Coulter). An equal volume of nicotine was mixed with the test compounds, resulting in a final screening concentration of 10 μM for the test compounds, and an EC_90_ concentration of nicotine for each subtype tested. Internal zero- and positive-signal controls were included in all plates, namely DMSO-only and EC_90_ nicotine (12 replicates at 0.2% (v/v) to correspond with the test compounds). Mecamylamine, a known, poorly subtype-selective nAChR antagonist serving as an antagonist control, was also included in each plate in a 12-point, 3-fold concentration response format as a control antagonist, in the presence of EC_90_ nicotine. The resulting final assay concentrations of these mecamylamine controls were in the range of 100 μM to 0.56 nM.

### Membrane potential screening

For the SH-EP1-*α*4 *β*2-nAChR and SH-EP1-*α*6/3 *β*2*β*3-nAChR lines: cells were seeded into 384-well black tissue culture plates (Corning #3712) at 7000 cells per well in 50 μl assay media using a microfill (BioTek). The plates were incubated overnight at 37 °C to allow cells to adhere to the plate, then transferred to 29 °C and incubated for ~72h to maximize receptor expression. On the compound addition day, medium was aspirated and plates washed one time with wash buffer (1x HBSS with 20 mM HEPES adjusted to pH 7.4) using a BioTek ELx405 Select CW Plate Washer. Twenty-five microliters of 0.5x FLIPR Membrane Potential dye (Molecular Devices, R8034; prepared in wash buffer) was added to the plates using a microfill. The plates were further incubated at 29 °C for 60 min and background fluorescence was measured (Envision, Perkin Elmer). An Acoustic Transfer System (ATS-100, EDC Biosystems Screen) was used to dispense 50 nl of the compound library supplemented with nicotine, or controls, into each well. Due to similarities between the nicotine concentration-response response range for the *α*4*β*2- and *α*6/3 *β*2*β*3-nAChR subtypes, the compound library plates used the same EC_90_ nicotine concentrations (500 nM final assay concentration) for these two subtypes. Plates were then incubated at room temperature for 20 min and fluorescence was measured again. Background fluorescence was subtracted from the final fluorescence read during data analysis.

For the SH-EP1-*α*3*β*4-nAChR cell line, procedures were the same as outlined in the preceding paragraph, except that after addition of nicotine (20 μM final assay concentration) and test compounds, the incubation temperature was 29 °C instead of room temperature.

### Quality control (QC) criteria

During assay development and optimization, as well as the HTS, assay sensitivities and robustness were evaluated using the following parameters: CV of controls, S:B ratio, and Z’-factor values. EC_50_ values were calculated using nonlinear regression analysis via GraphPad Prism 7 (GraphPad software, Inc.).

### Library screening data analysis

After subtracting background fluorescence from fluorescent read for each plate well, the raw data were converted into a list and all plates were appended by run. For analysis purposes, each data point was annotated with well coordinate, well type, compound identifier, titer plate barcode, run identifier, date, and protocol information. Basic statistics and QC criteria were calculated within TIBCO Spotfire (v7.0.0). Basic statistics of control wells were examined by well type, by run, plate, and row/column to monitor run-to-run and plate-to-plate variation, and to exclude possible experimental issues such as striping. After outlier removal and upon satisfactory QC, the data were normalized to internal plate controls (DMSO-only and EC_90_ nicotine). Concordance between replicates was assessed using linear regression. For each test well, the percentage of inhibition was obtained by subtracting the normalized fluorescent signal (NFS), expressed as a percentage, from 100%. Hit selection thresholds were defined from NFS distributions for test wells in each run, as those wells with NFS lower than the average NFS minus 3 standard deviations (SD).

### Orthogonal validation screen (^86^Rb^+^ Assay)

Orthogonal assays used ^86^Rb^+^ efflux, a direct and highly selective measurement of ion-flux through the nAChR channel [27], to validate the nAChR activity of hits identified in the primary screen. Compounds that did not replicate their behavior between the primary and orthogonal screens were designated as non-nAChR-mediated.

Cells were grown to confluence in in 24-well plates (Falcon; 500 *μ*l volume per well). The evening before each assay was to be performed, the medium was removed and replaced with 250 *μ*l per well of complete medium supplemented with ~350,000 cpm of ^86^Rb^+^ (NEN; counted at 40% efficiency using Cerenkov counting and the Packard TriCarb 1900 Liquid Scintillation Analyzer). This allowed overnight loading of cells with tracer. The assay was based on a proven, routine ^86^Rb^+^ efflux assay “flip-plate” protocol that includes 1500 nM atropine to exclude possible contributions of muscarinic acetylcholine receptors [27], with test compound or control solutions applied for 30 s.

Determinations of antagonist efficacy were performed in triplicate in each plate, and each antagonist was tested in three separate experiments. For each experiment, in one triplicate set of control samples, total ^86^Rb^+^ efflux was assessed in the EC_90_ concentration of carbamylcholine only, while non-specific ^86^Rb^+^ efflux was determined in another set of triplicate control samples (either in the presence of an EC_90_ concentration of carbamylcholine plus 100 *μ*M mecamylamine, which fully blocked agonist-induced and spontaneous nAChR-mediated ion flux, or in the presence of efflux buffer alone). Both determinations of non-specific efflux were equivalent. Specific efflux was then taken as the difference in control samples between total and non-specific ^86^Rb^+^ efflux, and was used to calculate the efficacy of blocking specific efflux by the test compounds.

## Results

### Validation of nicotine signals

We used the SH-EP1 cell line to host stably-transfected, defined, nAChR subtypes. SH-EP1 is well-suited to this general approach because, as noted in the Introduction, it does not express nAChR genes itself, but is permissive for functional expression of introduced nAChR subtypes. All nAChRs are ligand-gated ion channel receptors, permeable to K^+^ and Na^+^ ions; their activation depolarizes the resting electric potential across the cell membrane [28] and makes membrane potential assays a measure of function with broad prospective applicability across multiple nAChR subtypes.

**Fig. 1.**
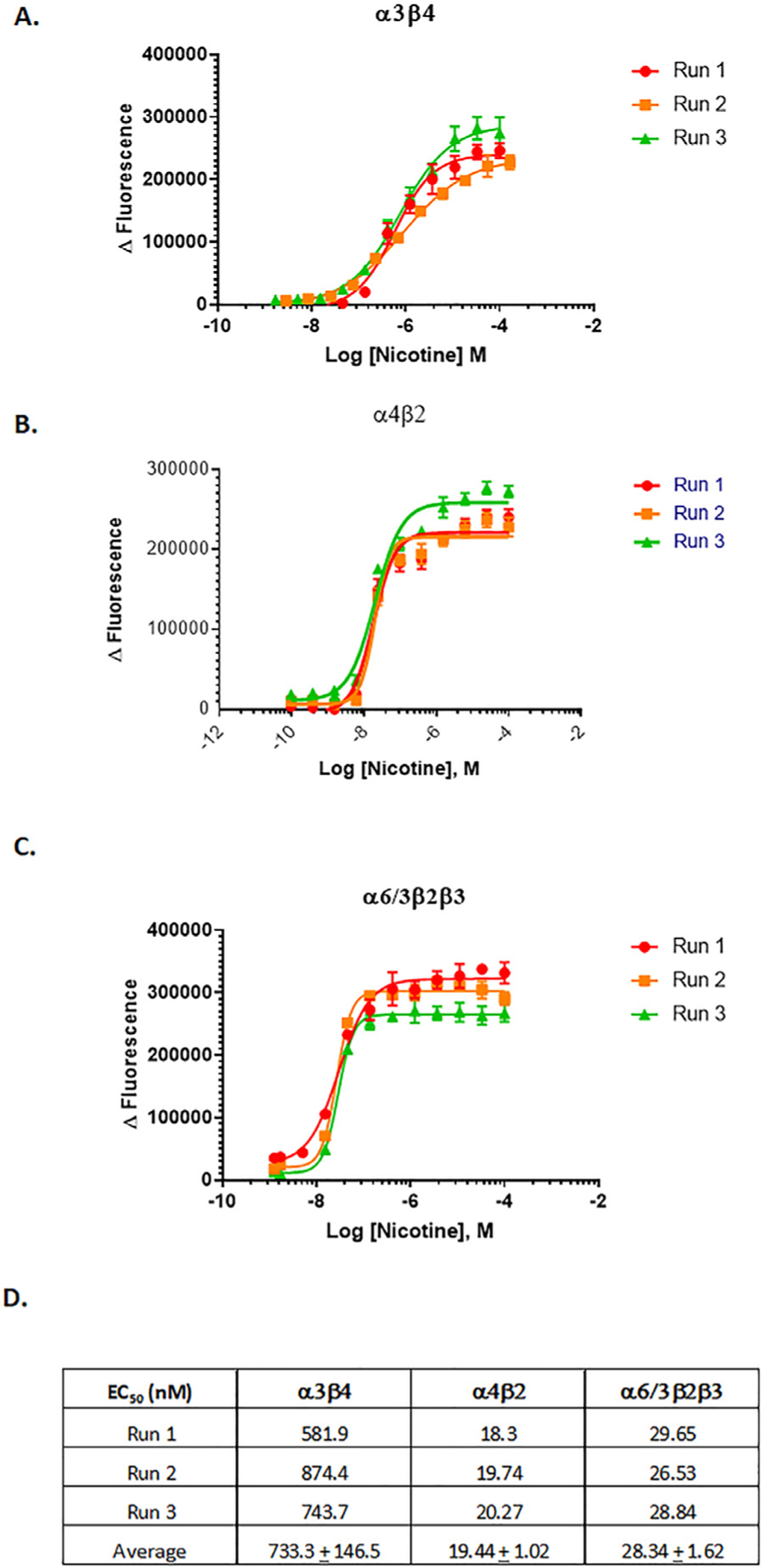
Nicotine induction verification in membrane potential assay. SH-EP1 cells expressing nAChR subtypes **(A)** *α*3*β*4, **(B)** *α*4*β*2, or **(C)** *α*6/3 *β*2 *β*3 were seeded at 7000 cells per well and treated with a 12-point, 1:3 serial dilution from 100 μM of (−)-nicotine. Concentration-dependent response curves were achieved in three independent experiments on different days. Each data point represents mean ± SD (four replicates per condition/concentration). Curve fitting was performed using nonlinear regression four-parameter methods from GraphPad Prism 7, and EC_50_ values are summarized in **(D)**.

To verify that nAChR function can be detected using a membrane potential assay, we initially determined nicotine concentration-response curves for all three nAChR subtypes of interest (*α*3*β*4, *α*4*β*2, and *α*6 *β*2). Three separate runs of nicotine concentration-response curves were determined for each subtype expressed in SH-EP1 cells, which showed high reproducibility of EC_50_ and maximal signal over baseline (Δ Fluorescence; Fig. 1 A–C). The rank order of potency based on mean EC_50_ values was *α*4*β*2 ≈ *α*6/3 *β*2*β*3 > *α*3*β*4 with mean EC_50_ values of 19.44 ± 1.02, 28.34 ± 1.62, and 733.3 ± 146.5 nM respectively (Fig. 1 D). This rank order of potency is consistent with long-standing results from two-electrode voltage-clamp electrophysiology experiments [29, 30].

### Assay optimization for standard agonist conditions

We worked extensively to optimize assay performance and thereby establish assays that would be robust and reproducible at high-throughput scale. Antagonists would have to be identified by their ability to reduce an agonist-induced signal. Accordingly, we began by identifying conditions that produced robust and reproducible agonist responses. All assay optimizations were performed identically across all three defined-subtype-expressing cell lines; here, we discuss the details associated with the SH-EP1-*α*4 *β*2-nAChR line as an example.

First, because all the compounds in the screening library were dissolved in DMSO, we tested each cell line’s tolerance for DMSO. We tested a range of DMSO concentrations (10%–0.25% v/v) in the presence of nicotine induction, then measured the effects on absolute fluorescent membrane potential dye signal magnitude, as well as cell viability. Our results indicated that DMSO had no significant effect on fluorescent signal window or cell viability under any of the conditions tested, except for the highest concentration of DMSO (10%), at which the fluorescent signal increased and cell viability decreased drastically. However, our final HTS conditions produce an in-assay DMSO concentration of only 0.2% (v/v) using an acoustic dispenser. This easily avoids any potential cell toxicity or false signals caused by high concentrations of DMSO.

We next optimized the membrane potential dye pre-incubation time. This step was needed because baseline signal reads are taken before compound addition, which allows compound-induced changes in signal magnitude to be defined precisely. Our data indicated that the background fluorescent signal reached a steady state at 20–80 min after dye loading. We chose to standardize to 60 min after loading the dye before taking the first (baseline signal) read, because this time point falls comfortably within the stable time-frame of 20–80 min and allows us to proceed with maximum throughput.

Having established basic working conditions, we addressed additional parameters to optimize assay performance: cell seeding density (varied from 4000–8000 cells per well in a 384-well plate) and the time of compound incubation time (compound addition immediately followed the baseline signal read; every 5 min from 5–30 min post-addition). At each optimization step, we determined standard nicotine concentration-response curves and evaluated the assay’s performance using the following assay parameter evaluations: CV of controls, S:B ratio, and Z’-factor of the assay. Z’-factor is a statistical measure of assay quality that includes both variability and signal dynamic range, indicating an assay’s robustness and reliability [31]. As expected, at each time point, the signal increased as the cell seeding density increased (Supplementary Figure 1). The resulting nicotine concentration-response curves were very consistent across various seeding densities and nicotine induction times, with EC_50_ values calculated using GraphPad Prism all closely falling around a mean of ~14 nM (Supplementary Figure 1). Importantly, Z’-factor values also increased dramatically as the seeding density increased (Supplemental Table 1), hitting a maximum at the condition of 7000 cells per well. Although we ideally would have preferred to use conditions providing the highest signal (8000 cells/well), 7000 cells/well gave the highest signal-to-noise ratio as well as the lowest CV values (Supplemental Table 1). For these reasons, we chose 7000 cells per well as the optimal seeding density. Data shown in Supplemental Table 1 also revealed that both 15 min and 25 min post-compound readings produced optimal QC performances, when using a seeding density of 7000 cells per well. Considering our finding that the fluorescent signal of membrane potential dye is only stable for 80 min, and that we pre-incubate the dye for 60 min before adding test compounds, therefore, a 15-min compound incubation time was chosen as the optimal condition to ensure the compound-induced signal is read within the 80-min window.

Having established optimal working parameters for the assay, we continued to fine-tune additional parameters. In each case, conditions that had a more favorable CV, S:B ratio, and Z’-factor were chosen as part of the assay optimization. As noted in [24], incubation of nAChR-expressing cell lines at lower temperatures (29°C) can sometimes increase the expression of nAChRs, and therefore could increase signal magnitude. Our data suggests satisfactory QC parameters (CV values, S:B ratio and Z’-factor) were met following either 48 h or 72 h incubation at 29°C; with Z’-factors slightly more robust at 72 h. Our fluorescent plate reader allows acquisition of measurements using either a slow- or fast-flash method. QC parameters were similar in either case; therefore, we chose fast-flash as the optimal method because it provided a slight advantage for HTS. We also optimized our assay by varying components of the cell growth medium (absence or presence of phenol red and various serum conditions) as well as temperature of incubation.

Once the assay conditions were finalized, we re-tested nicotine response curves dozens more times to establish a reliable EC_90_ concentration, for each nAChR subtype, to be used in the antagonist screens. Furthermore, we repeatedly tested whole 384-well microtiter plates treated only with EC_90_ nicotine or DMSO. This showed us that there was a plate-edge effect (data not shown); accordingly, we chose to eliminate the use of wells found within 2 rows or 2 columns from the plate edge for the compound library screen.

### Antagonist validation and positive controls

**Fig. 2.**
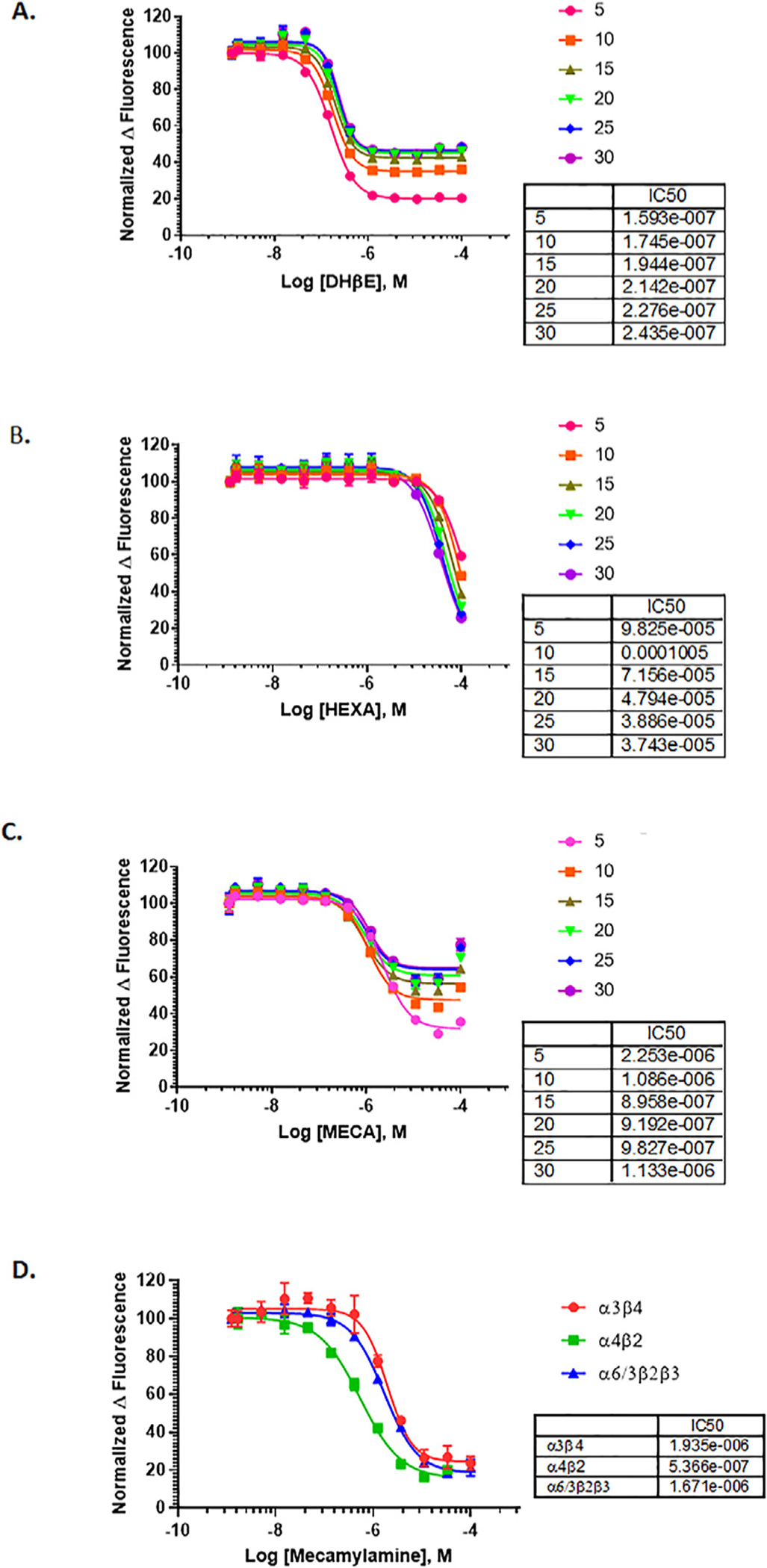
Validation of known nAChR antagonists. SH-EP1-*α*4 *β*2-nAChR cells were treated in a concentration-dependent manner with three known nicotine antagonists, **(A)** Dh *β*E, **(B)** hexamethonium, or **(C)** mecamylamine, in the presence of 500nM of nicotine. Data are shown for various incubation times, and all data were normalized to DMSO control. **(D)** Cells expressing different subtypes were treated with a range of mecamylamine concentrations serially diluted from 100 μM to 0.56 nM in the presence of nicotine at the pre-determined EC_90_ value (20 μM for *α*3*β*4 and 500 nM for *α*4*β*2 and *α*6/3 *β*2 *β*3). All data are presented as means +/− SD of quadruplicate wells (n = 4). Curves were fitted using nonlinear regression four parameter methods from GraphPad Prism 7 and IC_50_ values (M) are summarized as table inset for each antagonist at individual time point and/or receptor.

Once standard agonist conditions were established, we next performed initial antagonist activity validation of the three nAChR subtypes using established antagonists. These were the competitive antagonist dihydro-*β*-erythroidine (DH *β*E) [32], and a pair of non-competitive antagonists (hexamethonium and mecamylamine) [33]. We only describe SH-EP1-*α*4 *β*2-nAChR as an example here. Signal reads were taken at 5 min intervals after compound addition together with nicotine at EC_90_ (5–30 min range). For each subtype, IC_50_ values of the antagonists were very similar across all time-points examined for each antagonist (Fig. 2). Because of this, we averaged values across all time-points for each combination of compound and subtype that we examined. For the *α*4*β*2-nAChR used as an example here, DH *β*E and mecamylamine (average IC_50_ values = 0.20 ± 0.03 μM and 1.21 ± 0.52 μM, respectively) both showed potent antagonist activity, whereas hexamethonium (average IC_50_ value of 65.8 ± 28.8 μM) elicited responses only at high concentrations (Fig. 2 A–C). The rank order of potency for these inhibitors was DH *β*E > mecamylamine > hexamethonium, an observation consistent with a previous finding using an electrophysiological approach [34]. We further compared the response to the noncompetitive inhibitor mecamylamine across all three nAChR subtypes (Fig. 2D). The rank order of potency was similar: *α*4*β*2 > *α*6/3 *β*2*β*3 ≈ *α*3*β*4, with average IC_50_ values of 0.54, 1.67, and 1.91 μM respectively. Mecamylamine, along with nicotine, were included as internal controls in each screening plate in our compound library screening, which allowed us to monitor nicotine activity, normalize data, and evaluate data quality for each assay run.

### Identification of subtype-specific antagonists

Next, we performed a pilot screen to assess the feasibility of using the optimized nicotine membrane potential assay to identify antagonists of nAChR function, including subtype-selective antagonists. For this purpose, we used the Prestwick chemical library, which includes 1200 compounds, supplemented with a collection of 1098 CNS-targeted discovery compounds. The screen was conducted in 384-well format using a robotic platform. In brief, library compounds were premixed with nicotine at EC_90_ concentration for each subtype (20 μM nicotine for *α*3*β*4, and 500 nM nicotine for *α*4*β*2 and *α*6/3 *β*2*β*3). An acoustic dispenser was used to add the compounds, together with nicotine, into assay plates containing each nAChR subtype-expressing SH-EP1 cell line, yielding a final assay concentration of test compound at 10 μM and a final DMSO concentration of 0.2%. We performed antagonist screens in duplicate for all three nAChR subtypes: *α*3*β*4, *α*4*β*2, and *α*6/3 *β*2*β*3.

**Fig. 3.**
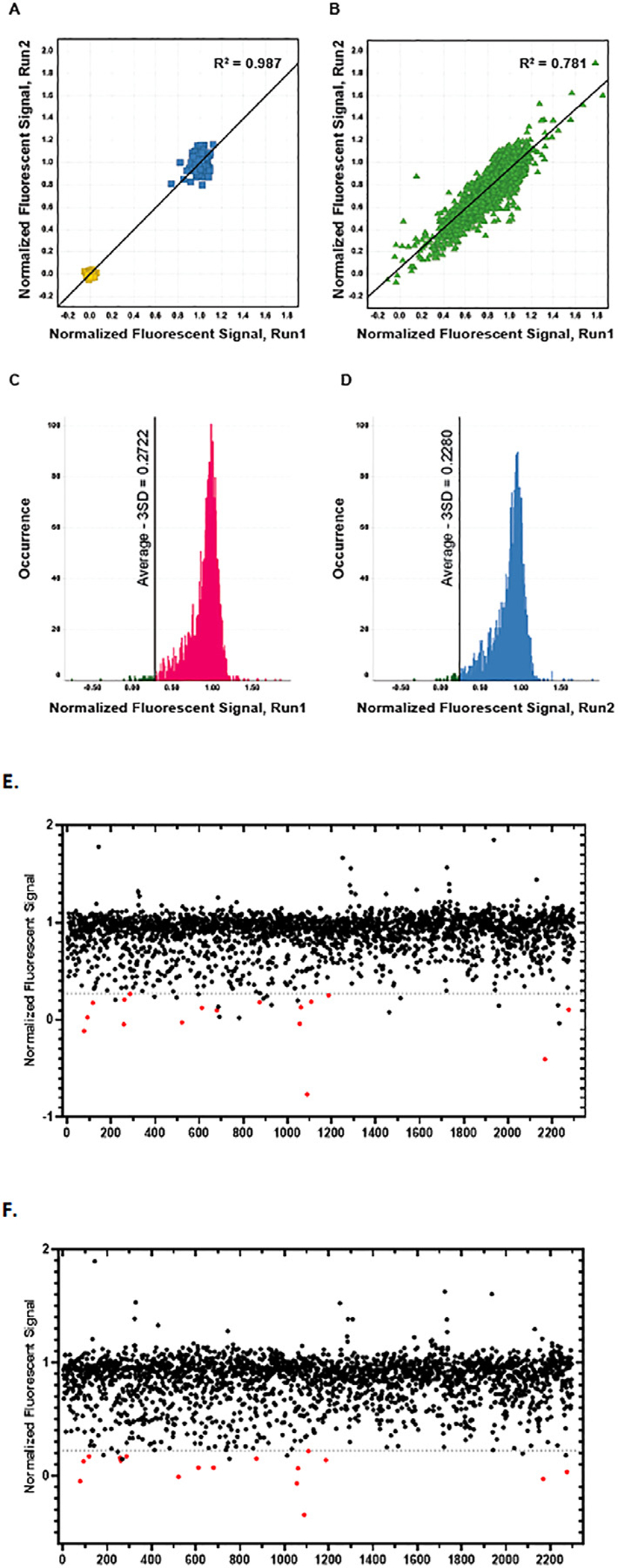
HTS results for SH-EP1-*α*4 *β*2-nAChR. Raw fluorescent signals acquired during two independent HTS runs were normalized to positive-signal (nicotine EC_90_) and zero-signal (DMSO) internal plate controls. The normalized fluorescent signals (NFS) were analyzed for run-to-run reproducibility and selection of hits to be advanced in orthogonal assays. **(A, B)** Scatter plots of the NFS of two independent HTS runs in SH-EP1 cells expressing human *α*4*β*2, evaluating assay reproducibility. Please note that data from internal controls were plotted on a separate panel **(A)**, adjacent to those from test compounds **(B)**, to avoid extensive overlap and thus crowding. HTS results were highly reproducible as evaluated for **(A)** internal positive controls (antagonist mecamylamine in EC_90_ of nicotine, yellow) and negative controls (DMSO in EC_90_ of nicotine, blue) (R^2^ = 0.987) and for **(B)** test compounds from library (green triangles, R^2^ = 0.781). **(C, D)** Occurrence histograms capturing the distribution of NFS in test wells for run 1 (**C,** red) and run 2 (**D,** blue) respectively. Hit selection thresholds (mean – 3 SD) are marked as vertical lines on either plot. Selected hits are represented as green histogram bars. **(E,F)** Scatterplots of the NFS as a function of the test compounds for HTS run 1 **(E)** and run 2 **(F)** are presented, each point represents a single compound. Please note that internal control values are not shown on these panels since they would reduce clarity. The no antagonist control value (baseline) is, by definition, 1 in these plots while the full agonist control value is, by definition, 0. The plots show clusters of signals at baseline (NFS = 1) for all inactive compounds. The hit selection threshold for this run (mean – 3 SD) is marked as a horizontal dotted grey line. Red dots with NFS values below the selection threshold indicate the overlapping hits from both runs, which were further tested in orthogonal assays.

We first evaluated the data resulting from these screens using QC parameters (CV, S:B, and Z’-factor). The outcomes are summarized in Table 1. In brief, CVs ≤ 10%, S:B ratios > 60, and Z’-factors > 0.74 were achieved, indicating that we had established robust nAChR assays. We present assay replication data for the SH-EP1-*α*4 *β*2-nAChR assay as an example in Fig. 3, with equivalent data provided for the remaining two screens in Supplemental Figure 2. We observed high reproducibility for the controls included in each screen, with mean R^2^ >0.99 across all three screens. For test compounds, reproducibility was also very high (e.g., the worst R^2^ value recorded for any test compound was 0.724). Fluorescence values in the presence of test compounds were normalized using the mean values of the negative and positive signal controls included in each plate. We considered compounds that reduced the signal evoked by > 3 SD compared to the mean normalized positive control signal to be hits (illustrated in Fig. 3C,D for SH-EP1-*α*4 *β*2-nAChR; equivalent data are shown for the other two subtypes in Supplemental Figure 3). The numbers of hits we identified in each of the primary screens were (Table 2):

SH-EP1-*α*3*β*4-nAChR: 77 unique hits in either run, of which 36 were found in both runs.
SH-EP1-*α*4 *β*2-nAChR: 41 unique hits in either run, of which 17 were found in both runs.
SH-EP1-*α*6/3 *β*2*β*3-nAChR: 53 unique hits in either run, of which 29 were found in both runs.

### Validation of hits, subtype-specific inhibitors

We selected compounds identified as hits in both runs of each of the primary screens to be advanced for validation using an orthogonal assay (^86^Rb^+^ efflux). We were unable to resupply only a few of the selected compounds. The numbers of advanced hits we were able to resupply were 35 of 36 for SH-EP1-*α*3*β*4-nAChR, 16 of 17 for SH-EP1-*α*4 *β*2-nAChR, 29 of 29 for SH-EP1-*α*6/3 *β*2*β*3-nAChR (Table 2).

As noted in the methods section, the ^86^Rb^+^ efflux assay is well established as a direct and highly selective measure of nAChR function. Z’-scores were calculated for each of our ^86^Rb^+^ efflux assays, using the data from the agonist-only and non-specific efflux control wells present in triplicate on each plate. The resulting Z’-scores were 0.88 ± 0.06 for *α*3*β*4, 0.78 ± 0.09 for *α*4*β*2, and 0.75 ± 0.11 for *α*6/3 *β*2*β*3, indicative of excellent assay quality. Compounds identified in the initial antagonist screen as potential hits were classified as “validated” if they reduced specific efflux by > 3x SD compared to no-drug control values. Validation rates were 97% (34 of 35 resupplied compounds for the SH-EP1-*α*3*β*4-nAChR assay), 63% (10 of 16 resupplied compounds for the SH-EP1-*α*4 *β*2-nAChR assay), and 59% (17 of 29 resupplied compounds for the SH-EP1-*α*6/3 *β*2*β*3-nAChR assay) (Table 2). The mean validation rate across all three assays was 73%, which is high considering the hits were validated using an orthogonal assay. This further confirms/validates our cell-based assays as reflecting true nAChR activity.

**Fig. 4.**
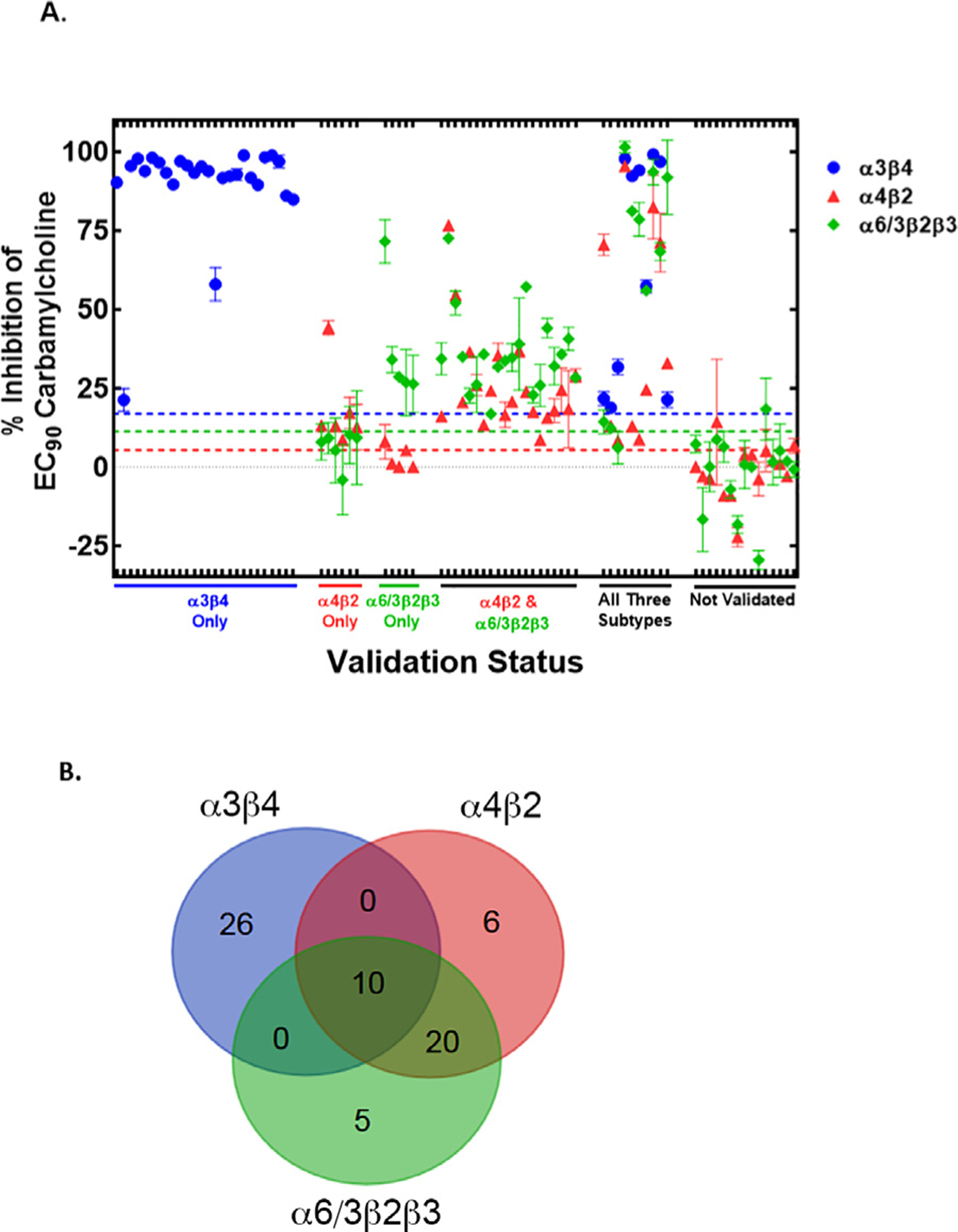
Validation and specificity testing of HTS antagonist hits using an orthogonal assay. SH-EP1 cells stably expressing human *α*3*β*4-, *α*4*β*2-, or *α*6/3 *β*2 *β*3-nAChR were loaded with ^86^Rb^+^, pretreated for 5 min with compounds identified as antagonist hits in HTS, then exposed to an EC_90_ concentration of carbamylcholine for 3 min in the presence of the test compound. Atropine (1.5 μM) was included in all wells to block the potential function of muscarinic acetylcholine receptors that might otherwise be stimulated by carbamylcholine. **(A)** All plates were normalized to internal maximum and minimum controls, as described in the Materials and Methods section. The no-antagonist control values for each assay were used to define 0% inhibition of carbamylcholine-induced activity, while the full-antagonist control values were used to define 100% inhibition. Activity measured at *α*3*β*4-nAChR is shown with blue symbols, that at *α*4*β*2-nAChR with red symbols, and that at *α*6/3 *β*2 *β*3-nAChR with green symbols. Dashed lines are color-matched to data symbols and designate the 3 X SD thresholds above the no-antagonist controls, used to determine validation of antagonist activity in each of the assays. Compounds are grouped according to their validated activity across the three nAChR subtypes addressed by this study. **(B)** Venn Diagram presents overlap of validated hits across the *α*3*β*4-, *α*4*β*2-, and *α*6/3 *β*2 *β*3-nAChR subtypes.

To maximize the amount of antagonist activity data we could produce, we further tested all of the validated hit compounds across all three nAChR subtypes of interest, alongside further resupplied compounds that had appeared as potential hits in only one of the SH-EP1-*α*4 *β*2-nAChR or SH-EP1-*α*6/3 *β*2*β*3-nAChR assays. Addition of these further compounds from the two *β*2*-nAChR assays brought the total number of *β*2*-nAChR test compounds to 39, very similar to the total number of *α*3*β*4-nAChR test compounds (the 34 validated hits from the SH-EP1-*α*3*β*4-nAChR assay). The results of this more extensive testing are shown in Fig. 4, where the compounds are clustered based on their inhibition activity on one or more subtypes or groups of subtypes.

Beginning at the left of Fig 4 A, we identified many compounds that were selective antagonists of *α*3*β*4-nAChR. These 26 compounds are color-coded in blue, and located above the validation cutoff line (defined as inhibition > 3SD compared to the no-antagonist internal controls included in the *α*3*β*4-nAChR ^86^Rb^+^ assays; dashed blue line). Strikingly, 24 of these 26 *α*3*β*4-nAChR-selective compounds were highly efficacious (> 75% inhibition of nicotine-induced function) at the test concentration of 10 μM. Presumably, the overall very high efficacy at *α*3*β*4-nAChR underlies the exceptionally high validation rate we observed for this subtype.

Next, we saw a group of 6 compounds that were selective antagonists of *α*4*β*2-nAChR (validation cutoff line defined as inhibition > 3SD compared to the no-antagonist internal controls included in the *α*4*β*2-nAChR ^86^Rb^+^ assays; dashed red line). These are denoted as “*α*4*β*2 Only” in Fig 4A, and comparisons are shown to their lack of activity in the *α*6/3 *β*2*β*3-nAChR validation assay (green points, which fall below their corresponding validation cutoff line defined as inhibition > 3SD compared to the no-antagonist internal controls included in the *α*6/3 *β*2*β*3-nAChR ^86^Rb^+^ assays; dashed green line). A similarly small group of 5 compounds was identified that were selective antagonists of *α*6/3 *β*2*β*3-nAChR, named as “*α*6/3 *β*2*β*3 Only” in Fig 4 A. Note, lack of activity of these compounds at *α*3*β*4-nAChR is not displayed in order to reduce clutter near the 0% inhibition line for these two groups. This makes it easier to visualize their activity at *α*4*β*2- *vs*. lack of activity at *α*6/3 *β*2*β*3-nAChR (“*α*4*β*2 Only”), or *vice versa* (“*α*6/3 *β*2*β*3 Only”).

In contrast, a larger group of 20 compounds was identified that were validated antagonists of both *α*4*β*2- and *α*6/3 *β*2*β*2-nAChR subtypes, but not at *α*3*β*4-nAChR (named “*α*4*β*2 & *α*6/3 *β*2*β*3” in Fig 4 A, B). Interestingly, the majority of validated hits for the two nAChR subtypes containing the *β*2 subunit (*α*4 *β*2- and/or *α*6/3 *β*2*β*3-nAChR) were typically less efficacious (≤ 50% reduction of nicotine-induced function at the 10 μM test concentration), which contrasts with the previously noted high efficacy of compounds validated to be selective antagonists of *α*3*β*4-nAChR. Of further interest, the total number of compounds in the “*α*4*β*2 & *α*6/3 *β*2*β*3” group (20) is nearly double that of the combined total that fell into the “*α*4*β*2 Only” and “*α*6/3*β*2*β*3 Only” groups (5 + 6 = 11).

The final two groups identified are a set of 10 with activity at “All Three subtypes” (Fig 4A,B), and a set of 15 for which activity was not “Not Validated” (Fig 4A).

## Discussion

In this study, we demonstrated HTS-suitable, cell-based functional assays for the three major heteromeric nAChR subtypes known to play defined roles in the initiation and maintenance of smoking behavior, *α*3*β*4*-, *α*4*β*2*-, and *α*6 *β*2*-nAChR. Each of the cell lines produced to express these smoking-relevant subtypes was engineered using the same SH-EP1 cell background, which does not itself express nAChR subunits [23]. The major advantage of this approach is that it guarantees low/no background from the parental cell line. An additional significant advantage is that false-positives identified in any one screen can confidently be flagged as potentially problematic in the others.

The SH-EP1-*α*6/3 *β*2 *β*3-nAChR used a chimeric *α*6/3 nAChR subunit. This was composed of the N-terminal extracellular agonist binding domain of the *α*6-nAChR subunit fused to the remainder of the *α*3-nAChR subunit sequence, in order to obtain good functional expression in the cell line [24]. A possible caveat of using such chimeric subunits is that some compounds that interact allosterically (i.e., outside of the N-terminal competitive-agonist binding domain) might exhibit pharmacology resembling that of an *α*3*-nAChR. Fortunately, the amino acid sequences of *α*3- and *α*6-nAChR subunits share close sequence homology [29]. As a result, even for a non-competitive compound, differences are be expected to be subtle in most cases, and any potential leads can be definitively addressed using lower-throughput approaches that can heterologously express functional *α*6*-nAChR using subunits with entirely-natural protein sequences [35]. It is important to note that, in contrast to *α*6 *β*2*-nAChR cell lines previously used in HTS-suitable approaches [34, 36], this SH-EP1-*α*6/3 *β*2 *β*3-nAChR line does not rely on introduction of a “gain-of-function” mutation in the ion-channel domain of the receptor in order to produce robust function. Such mutations have been shown to affect receptor pharmacological profiles, even to the extent of changing competitive antagonist into agonist activity [37]. Thus, our assay avoids this potential pitfall of altered pharmacology due to the presence of a gain-of-function mutation.

All three cell-based nAChR assays were initially validated by assessing an appropriate response to nicotine as well as known antagonists. As noted in the Results, our observations were consistent with previously published studies. We further verified the assays by performing pilot compound library screening and profiling. Performance of the screens was excellent for all three assays as measured by Z-scores [38] (0.73–0.81) and very low signal CVs (0.024–0.10) across test wells, and the assays showed limited sensitivity to DMSO, the standard solvent used to prepare HTS compound libraries. These parameters indicate our functional assays are robust and reliable for identifying meaningful hits, and thus well suited to HTS applications. In addition, our assays can potentially be further miniaturized into a 1536-well format to reduce costs and increase throughput as needed. Advantages of our HTS approach over traditional assays (including recently published assays using increased-throughput patch-clamp electrophysiology [34, 39]) include the potential for higher-throughput with fluorescence-based primary screens, as used here; higher Z-scores and hit validation rates; no special requirements for equipment; and no use of radioactive material, all of which improve assay discrimination and are considered HTS-friendly characteristics.

Reflecting the excellent assay discrimination scores measured in this study, moderate hit rates were achieved (at < 1.20% across the three assays, see Table 2 adjusted hit rate, defined as compounds identified as active in both runs of a particular assay). Further, validation rates with orthogonal assays (^86^Rb^+^ efflux) of initial hits from the primary screens were excellent (ranging from 97% for *α*3*β*4-nAChR to 59% for *α*6 *β*2 *β*3-nAChR; mean of 73% across all three subtypes). This further validates our primary screening assays’ ability to identify compounds with authentic nAChR activity. Most compounds with validated *α*3*β*4-nAChR antagonist activity did not show activity at either of the *β*2*-nAChR subtypes, and *vice versa*. In contrast, cross-activity between the validated hits at *α*4*β*2- and *α*6 *β*2-nAChR was common. This outcome is consistent with the established understanding that nAChR hosting *β*2 subunits tend to have similar pharmacological profiles, which are quite different from those hosting *β*4 subunits, and that *β*-subunit complement can be equally, if not more, determinative of nAChR pharmacological profiles than that of *α*-subunits [30]. Results from the standard nicotinic compounds that we used as in-plate controls were also compatible with established nAChR pharmacology. Activation by nicotine, and antagonism by the competitive antagonist DH *β*E was more potent at the two *β*2-containing nAChR subtypes than at *α*3*β*4-nAChR [30]. The relative lack of subtype selectivity by the broad-spectrum non-competitive nAChR antagonist mecamylamine was also compatible with established literature findings [30].

Reassuringly, the hits found within the Prestwick library included mecamylamine, which was already included as a control compound in each of our assays and was identified and validated as a hit at all three subtypes. Most intriguingly, nortriptyline hydrochloride was also identified as a hit at the *α*3*β*4-nAChR. This finding is especially interesting since nortriptyline has been explored extensively as a smoking cessation drug [40]. It is tempting to speculate that, in addition to its intended activity as an antidepressant, its nAChR activity may contribute to its smoking cessation effects.

A major potential application of developing this assay panel is to determine which compounds, in addition to nicotine, may modify the addictive nature of tobacco products. The best-established evidence is for NNTAs, which appear to modify the actions of nicotine itself, or even substitute for it in low-nicotine tobacco products (summarized in the Introduction). There is also accumulating evidence that certain flavorants used in nicotine/tobacco products can have significant effects on nAChR function. Even within the test panel of ≈2000 compounds used in our pilot study, we identified several dozen compounds with significant activity at nAChR subtypes closely associated with smoking behavior. Accordingly, it is very likely that many additional nAChR-active tobacco compounds, including antagonists, remain to be discovered among the > 9000 other compounds [22] found in tobacco smoke. Our HTS assays can also be used to analyze the nAChR-mediated effects of the growing number of flavorants and additives being added to nicotine delivery devices and products, including “home brew” devices that are currently effectively unregulated. HTS is essential to performing this work in a timely, systematic, and consistent way, to allow the FDA to keep pace with, and regulate effectively, new products as they are devised.

Smoking-associated nAChR subtypes are also plausible targets for efforts to discover new smoking-cessation pharmacotherapies [41, 42]. The availability of high-quality HTS-capable assays and counter-screens will be invaluable in pursuing this objective. We note that the recently described automated patch-clamp assays [34, 39] would provide a superb alternative validation assay to our ^86^Rb^+^ efflux methodology in this context, providing greater throughout and removing the need to use radioactive materials.

Importantly, other abused substances also engage the withdrawal/aversion and reward/reinforcement pathways on which *α*3*β*4*-, *α*4*β*2*-, and *α*6 *β*2*-nAChR exert their effects. Accordingly, compounds identified using our novel assays (either within tobacco products, or in a search for novel smoking-cessation compounds) may have relevance to withdrawal/aversion and reward/reinforcement in alcohol consumption behavior and dependence [43–46], cocaine reward [47], and reward reinforcement in general [48]. More directly, our panel of HTS cell-based nAChR assays will be very valuable for identifying compounds in both combusted and non-combusted tobacco products that may have relevance to their use, with potential for regulation under the FSPTCA.

## Supplementary Material

Supplementary Data

## Figures and Tables

**Table 1:** HTS QC summary.

Receptor Subtype	CV range	Avg S:B value	SD (S:B value)	Avg Z’-factor value	SD (Z’-factor)	N (Plates)	# Evaluable compounds	% Data passed QC
***α*3*β*4**	**0.0249 – 0.0946**	147.848	42.006	0.810	0.054	24	2258	98.26%
***α*4*β*2**	**0.0243 – 0.0849**	61.458	16.786	0.789	0.050	24	2294	99.83%
***α*6/3*β*2*β*3**	**0.0454 – 0.104**	63.838	24.808	0.737	0.044	24	2294	99.83%

**Table 2:** HTS hits selection summary.

Receptor Subtype	# Unique hits (-3SD)	Primary screen hit rate	# of hits in both runs	Adjusted hit rate	# of hits resupplied for validation	# of validated hits	Validation rate
***α*3*β*4**	77	3.41%	36	1.594%	35	34	97.14%
***α*4*β*2**	41	1.79%	17	0.741%	16	10	62.50%
***α*6/3*β*2*β*3**	53	2.31%	29	1.264%	29	17	58.62%

## Abbreviations:

CNS: Central nervous system
CV: coefficient of variation
DH *β*E: dihydro-*β*-erythroidine
nAChR: nicotinic acetylcholine receptor
FDA: Food and Drug Administration
FSPTCA: Family Smoking Prevention and Tobacco Control Act
HTS: high-throughput screening
NFS: normalized fluorescent signal
NNTAs: non-nicotine tobacco alkaloids
S:B: signal to background ratio
SD: standard deviations
QC: quality control
