# Supplementary material for: High-throughput cell-based assays for identifying antagonists of multiple smoking-associated human nicotinic acetylcholine receptor subtypes
Source: SLAS Discov. Author manuscript; Available in PMC 2022 Feb 5. (PMC8816891; doi:10.1016/j.slasd.2021.10.001)
Supplement: Supplementary Data [file NIHMS1774558-supplement-Supplementary_Data.pdf]

Supplemental Materials

Title: High-throughput cell-based assays for identifying antagonists of multiple smoking-associated human nicotinic acetylcholine receptor subtypes

Michelle Kassner[a,$], J. Brek Eaton[b,$], Nanyun Tang[a], Joachim L. Petit[c], Nathalie Meurice[c], Hongwei Holly Yin[a,#,*] hoyin@coh.org., Paul Whiteaker[b,*] Paul.Whiteaker@BarrowNeuro.org

[a]Cancer and Cell Biology Division, Translational Genomics Research Institute, Phoenix, AZ 85004, USA

[b]Division of Neurobiology, Barrow Neurological Institute, St. Joseph's Hospital and Medical Center, 350 W. Thomas Rd., Phoenix, AZ 85013, USA

[c]Department of Hematology/Oncology, Mayo Clinic, Scottsdale, AZ 85259, USA

[*]Corresponding authors

[$]These authors contributed equally

[#]Current Address: Beckman Research Institute of City of Hope, 1500 E Duarte Road, Duarte, CA 91010, USA

A short title (up to 45 characters): HTS assays for multiple human nAChR subtypes

Keywords (3-5):

nicotinic acetylcholine receptor, membrane potential assays, cell-based screening

Supplemental Table 1. Quality Control (QC) evaluation of α4β2 assay optimization.

		CV					
	Nicotine[nM]	5	10	15	20	25	30
4000	100000.00	4%	4%	4%	6%	5%	7%
	25000.00	5%	5%	5%	5%	7%	6%
	6250.00	6%	7%	7%	7%	8%	8%
	1562.50	8%	8%	7%	7%	7%	7%
	390.63	8%	9%	10%	11%	11%	12%
	97.66	9%	10%	10%	11%	12%	13%
	24.41	4%	5%	6%	7%	6%	8%
	6.10	6%	8%	11%	13%	14%	16%
	1.53	16%	23%	28%	24%	28%	26%
	0.38	13%	9%	10%	14%	14%	16%
	0.10	18%	18%	18%	16%	21%	19%
	DMSO	34%	27%	21%	20%	23%	24%
5000	100000.00	5%	5%	5%	5%	4%	5%
	25000.00	12%	12%	12%	13%	13%	13%
	6250.00	7%	7%	7%	7%	7%	7%
	1562.50	3%	5%	6%	5%	6%	6%
	390.63	6%	5%	6%	8%	8%	10%
	97.66	8%	9%	10%	10%	11%	12%
	24.41	12%	11%	13%	18%	14%	19%
	6.10	8%	10%	11%	12%	15%	17%
	1.53	18%	12%	17%	20%	20%	19%
	0.38	8%	9%	12%	9%	12%	6%
	0.10	11%	4%	3%	4%	6%	6%
	DMSO	26%	21%	26%	20%	27%	23%
6000	100000.00	4%	4%	3%	2%	2%	2%
	25000.00	2%	2%	3%	4%	4%	4%
	6250.00	4%	3%	4%	5%	4%	6%
	1562.50	6%	6%	7%	8%	8%	9%
	390.63	5%	5%	6%	8%	8%	9%
	97.66	7%	7%	8%	9%	9%	11%
	24.41	10%	10%	12%	15%	14%	16%
	6.10	8%	6%	9%	11%	7%	11%
	1.53	21%	10%	14%	26%	19%	28%
	0.38	15%	14%	16%	18%	17%	19%
	0.10	47%	24%	18%	16%	12%	14%
	DMSO	8%	12%	15%	7%	13%	9%
7000	100000.00	2%	1%	1%	1%	1%	1%
	25000.00	5%	5%	4%	4%	3%	3%
	6250.00	5%	4%	4%	4%	3%	4%
	1562.50	3%	3%	4%	4%	5%	4%
	390.63	3%	3%	2%	2%	2%	3%
	97.66	1%	1%	1%	2%	2%	2%
	24.41	2%	4%	4%	5%	5%	4%
	6.10	2%	1%	3%	2%	3%	2%
	1.53	12%	5%	5%	9%	7%	12%
	0.38	10%	9%	11%	12%	16%	14%
	0.10	18%	16%	20%	21%	20%	20%
	DMSO	19%	20%	21%	17%	16%	15%
8000	100000.00	2%	3%	3%	4%	4%	5%
	25000.00	2%	2%	3%	4%	4%	5%
	6250.00	3%	4%	4%	6%	6%	7%
	1562.50	3%	4%	3%	3%	3%	2%
	390.63	4%	5%	6%	6%	8%	7%
	97.66	3%	4%	4%	3%	6%	4%
	24.41	5%	6%	5%	6%	7%	7%
	6.10	6%	8%	9%	9%	12%	10%
	1.53	15%	11%	16%	18%	19%	16%
	0.38	13%	10%	11%	11%	10%	9%
	0.10	11%	14%	13%	10%	11%	8%
	DMSO	12%	10%	9%	6%	8%	6%

		S:B RATIO					
	Nicotine[nM]	5	10	15	20	25	30
4000	100000.00	7.5	7.0	6.5	5.3	7.0	5.4
	25000.00	8.1	7.5	6.9	5.6	7.2	5.5
	6250.00	8.2	7.6	7.0	5.7	7.2	5.6
	1562.50	7.7	7.1	6.6	5.4	6.8	5.4
	390.63	6.9	6.4	5.9	4.9	6.1	4.9
	97.66	6.4	5.7	5.3	4.5	5.5	4.5
	24.41	5.6	5.1	4.7	3.9	4.8	4.0
	6.10	2.8	2.8	2.6	2.2	2.7	2.3
	1.53	0.9	1.2	1.3	1.3	1.5	1.4
	0.38	0.8	1.2	1.3	1.2	1.4	1.2
	0.10	0.9	1.0	1.0	1.2	1.2	1.2
	DMSO	1.0	1.0	1.0	1.0	1.0	1.0
5000	100000.00	6.5	6.7	6.6	4.9	7.1	4.9
	25000.00	6.1	6.2	6.1	4.7	6.4	4.5
	6250.00	6.8	7.0	7.0	5.2	7.2	5.0
	1562.50	6.7	6.7	6.6	5.2	7.0	5.1
	390.63	6.3	6.2	6.2	4.8	6.5	4.8
	97.66	5.7	5.5	5.5	4.4	5.7	4.3
	24.41	4.5	4.5	4.5	3.4	4.5	3.4
	6.10	2.7	2.9	2.9	2.3	2.9	2.3
	1.53	0.9	1.3	1.5	1.3	1.7	1.4
	0.38	0.7	1.1	1.3	1.2	1.4	1.2
	0.10	0.8	1.0	1.2	1.1	1.2	1.1
	DMSO	1.0	1.0	1.0	1.0	1.0	1.0
6000	100000.00	7.2	7.4	7.1	5.2	7.5	5.1
	25000.00	8.0	8.0	7.7	5.8	8.0	5.7
	6250.00	8.0	8.0	7.8	5.9	8.0	5.8
	1562.50	7.9	7.8	7.6	5.8	7.8	5.7
	390.63	7.3	7.2	7.0	5.4	7.3	5.4
	97.66	6.4	6.2	6.0	4.7	6.2	4.6
	24.41	5.5	5.5	5.3	4.1	5.4	4.1
	6.10	2.8	3.1	3.1	2.3	3.1	2.3
	1.53	0.8	1.3	1.4	1.2	1.6	1.3
	0.38	0.9	1.2	1.4	1.2	1.4	1.2
	0.10	1.2	1.5	1.6	1.4	1.6	1.4
	DMSO	1.0	1.0	1.0	1.0	1.0	1.0
7000	100000.00	7.4	8.1	8.0	6.2	8.8	6.4
	25000.00	8.2	8.8	8.6	6.9	9.4	7.1
	6250.00	8.1	8.6	8.5	6.9	9.2	7.0
	1562.50	7.9	8.3	8.2	6.8	8.9	6.9
	390.63	7.4	7.6	7.5	6.2	8.1	6.3
	97.66	6.7	7.0	6.9	5.7	7.3	5.8
	24.41	5.8	6.0	6.0	4.9	6.2	5.0
	6.10	2.8	3.4	3.4	2.9	3.6	2.9
	1.53	1.0	1.5	1.7	1.6	2.0	1.8
	0.38	0.9	1.3	1.5	1.4	1.6	1.4
	0.10	1.1	1.4	1.5	1.6	1.8	1.6
	DMSO	1.0	1.0	1.0	1.0	1.0	1.0
8000	100000.00	6.6	6.2	5.7	4.8	6.4	5.0
	25000.00	7.1	6.6	6.0	5.1	6.5	5.3
	6250.00	7.3	6.8	6.3	5.4	7.0	5.7
	1562.50	7.0	6.5	6.0	5.2	6.5	5.3
	390.63	6.7	6.1	5.7	5.0	6.2	5.2
	97.66	5.9	5.5	5.1	4.5	5.5	4.6
	24.41	5.2	4.8	4.5	4.0	4.7	4.0
	6.10	2.6	2.9	2.8	2.5	3.0	2.6
	1.53	1.0	1.4	1.5	1.5	1.8	1.6
	0.38	1.0	1.3	1.4	1.3	1.5	1.4
	0.10	1.0	1.1	1.1	1.2	1.2	1.2
	DMSO	1.0	1.0	1.0	1.0	1.0	1.0

		Z'-Factor					
	Nicotine[nM]	5	10	15	20	25	30
4000	100000.00	0.70	0.73	0.73	0.62	0.70	0.57
	25000.00	0.70	0.71	0.71	0.68	0.65	0.61
	6250.00	0.64	0.65	0.67	0.61	0.63	0.54
	1562.50	0.58	0.59	0.63	0.60	0.62	0.57
	390.63	0.55	0.54	0.52	0.42	0.46	0.37
	97.66	0.49	0.46	0.48	0.40	0.39	0.30
	24.41	0.62	0.62	0.60	0.50	0.58	0.44
	6.10	0.16	0.18	0.08	-0.17	-0.07	-0.38
	1.53	-18.35	-6.48	-4.65	-3.66	-2.71	-3.74
	0.38	-7.50	-6.07	-2.62	-3.77	-2.30	-4.53
	0.10	-14.20	-53.22	-29.99	-5.06	-7.39	-5.77
	DMSO						
5000	100000.00	0.66	0.72	0.69	0.66	0.73	0.65
	25000.00	0.43	0.44	0.41	0.34	0.40	0.30
	6250.00	0.60	0.64	0.62	0.59	0.64	0.56
	1562.50	0.76	0.72	0.66	0.68	0.66	0.62
	390.63	0.65	0.69	0.63	0.55	0.57	0.43
	97.66	0.55	0.53	0.46	0.44	0.43	0.32
	24.41	0.32	0.42	0.27	-0.03	0.21	-0.09
	6.10	0.13	0.24	0.07	-0.13	-0.11	-0.45
	1.53	-7.43	-2.49	-2.16	-3.26	-1.61	-2.95
	0.38	-2.44	-6.49	-3.37	-5.06	-2.16	-4.98
	0.10	-3.64	-48.06	-4.61	-4.53	-3.45	-5.74
	DMSO						
6000	100000.00	0.83	0.82	0.83	0.86	0.88	0.88
	25000.00	0.88	0.87	0.81	0.81	0.81	0.80
	6250.00	0.82	0.83	0.80	0.76	0.80	0.73
	1562.50	0.76	0.75	0.70	0.66	0.68	0.62
	390.63	0.79	0.78	0.73	0.65	0.66	0.59
	97.66	0.72	0.68	0.63	0.59	0.59	0.50
	24.41	0.56	0.57	0.46	0.31	0.41	0.26
	6.10	0.50	0.55	0.41	0.27	0.50	0.24
	1.53	-3.80	-1.43	-1.45	-4.20	-1.12	-3.37
	0.38	-3.77	-2.96	-2.00	-3.75	-1.54	-4.16
	0.10	-7.54	-1.98	-1.32	-1.21	-0.71	-1.25
	DMSO						
7000	100000.00	0.84	0.87	0.87	0.85	0.92	0.89
	25000.00	0.74	0.75	0.77	0.78	0.84	0.81
	6250.00	0.74	0.77	0.79	0.77	0.83	0.79
	1562.50	0.83	0.81	0.79	0.78	0.78	0.76
	390.63	0.80	0.80	0.82	0.82	0.87	0.82
	97.66	0.87	0.87	0.85	0.83	0.87	0.83
	24.41	0.80	0.75	0.71	0.67	0.74	0.72
	6.10	0.60	0.71	0.61	0.63	0.71	0.66
	1.53	-477.54	-0.61	-0.36	-0.54	0.12	-0.39
	0.38	-5.35	-2.14	-1.36	-1.70	-0.98	-1.53
	0.10	-16.20	-2.42	-1.92	-1.52	-1.01	-1.26
	DMSO						
8000	100000.00	0.86	0.84	0.83	0.82	0.81	0.77
	25000.00	0.87	0.86	0.83	0.81	0.82	0.78
	6250.00	0.83	0.81	0.80	0.75	0.76	0.71
	1562.50	0.83	0.81	0.82	0.85	0.85	0.87
	390.63	0.79	0.75	0.73	0.72	0.67	0.68
	97.66	0.82	0.78	0.78	0.82	0.74	0.78
	24.41	0.72	0.71	0.72	0.71	0.67	0.67
	6.10	0.47	0.48	0.44	0.45	0.35	0.42
	1.53	-59.10	-1.20	-0.98	-0.96	-0.58	-0.53
	0.38	-22.68	-1.48	-0.87	-0.87	-0.42	-0.44
	0.10	-12.69	-6.70	-4.62	-1.64	-2.07	-1.19
	DMSO						

Notes on highlight:

CV is highlighted in green if is <10% and yellow if is between 10 and 20%.

Signal to noise (S:B) ratio is highlighted in yellow if is between 5 and 10 folds.

Z'-factor is highlighted in green if is >0.7 and yellow if is between 0.5 and 0.699.

Supplemental Figure 1. Assay optimization using SH-EP1-α4β2-nAChR. Nicotine concentration-response curves in SH-EP1-α4β2-nAChR cells plated at various cell densities ranging from 4000-8000 per well in time-course modality. Cells were treated with serial dilutions of nicotine and data were acquired every 5 min from 5–30 min post-nicotine addition (light-protected between measurements). Corresponding nicotine induction curves and EC_{50} values were generated using nonlinear regression four-parameter methods from GraphPad Prism 7. The data are presented as means +/- SD of quadruplicate wells (n=4). EC_{50} values are summarized as table inset for each time point.

Supplemental Figure 2. Screening replication for SH-EP1-α3β4-nAChR and SH-EP1-α6β2β3-nAChR. Raw fluorescent signals acquired during two independent HTS runs for each subtype were normalized as described in detail in Figure 4. Scatter plots of the NFS evaluate assay reproducibility of two independent HTS runs in SH-EP1 cells expressing human α6β2β3-nAChR **(A-B)** and α3β4-nAChR **(C-D)**. HTS results were highly reproducible as evaluated for internal positive (yellow) and negative (blue) plate controls **(A, C)** and for test wells **(B, D)**.

Supplemental Figure 3. Hit selection for SH-EP1-α3β4-nAChR and SH-EP1-α6β2β3-nAChR. Raw fluorescent signals acquired during two independent HTS runs were normalized and analyzed as described in Figure 4. Hits with NFS below mean – 3SD in each individual run were selected. Occurrence histograms capturing the distribution of NFS in test wells are presented for SH-EP1 cells expressing human α6β2β3-nAChR **(A-B)** and α3β4-nAChR **(C-D)**. Data for run 1 are represented in red **(A, C)** and for run 2 in

blue **(B, D)**. Hit selection thresholds (mean – 3 SD) are marked as vertical lines; selected hits are represented as green histogram bars.

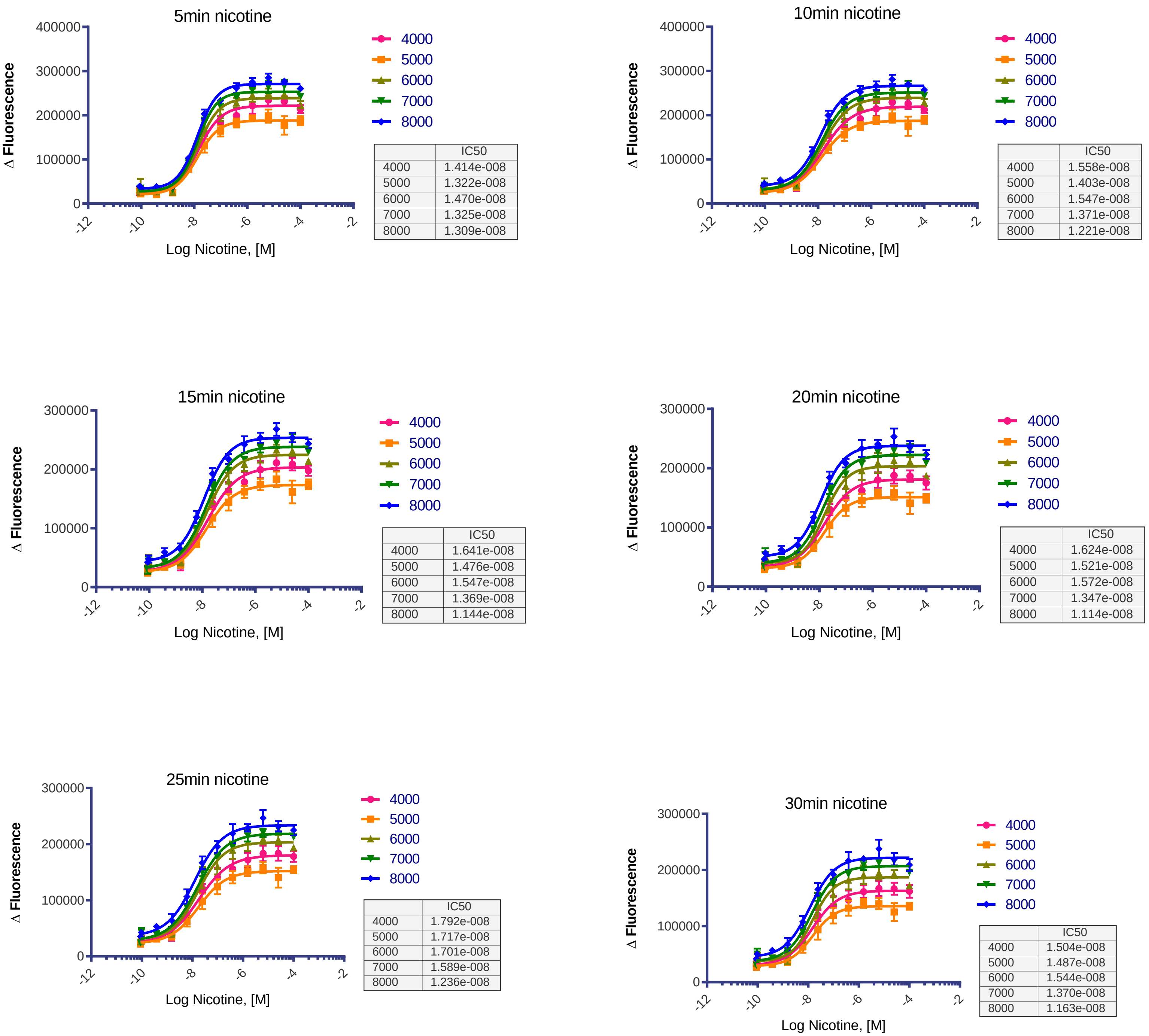

5min nicotine

	IC50
4000	1.414e-008
5000	1.322e-008
6000	1.470e-008
7000	1.325e-008
8000	1.309e-008

10min nicotine

	IC50
4000	1.558e-008
5000	1.403e-008
6000	1.547e-008
7000	1.371e-008
8000	1.221e-008

15min nicotine

	IC50
4000	1.641e-008
5000	1.476e-008
6000	1.547e-008
7000	1.369e-008
8000	1.144e-008

20min nicotine

	IC50
4000	1.624e-008
5000	1.521e-008
6000	1.572e-008
7000	1.347e-008
8000	1.114e-008

25min nicotine

	IC50
4000	1.792e-008
5000	1.717e-008
6000	1.701e-008
7000	1.589e-008
8000	1.236e-008

30min nicotine

	IC50
4000	1.504e-008
5000	1.487e-008
6000	1.544e-008
7000	1.370e-008
8000	1.163e-008

Supplemental Figure 1. Assay Optimization using SH-EP1-α4β2-nAChR

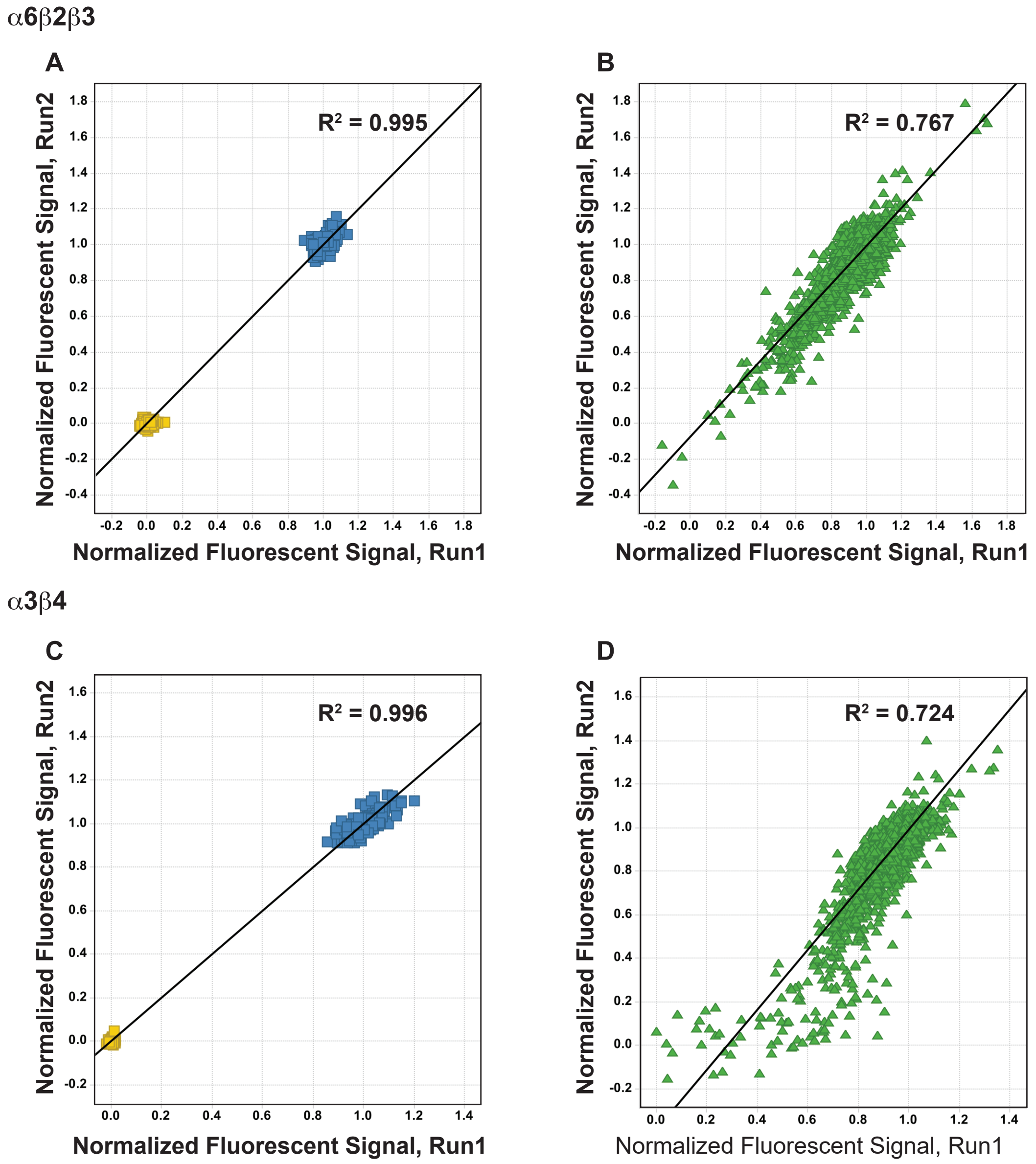

Supplemental Figure 2. Screening replication for SH-EP1-α3β4-nAChR and SH-EP1-α6β2β3-nAChR.

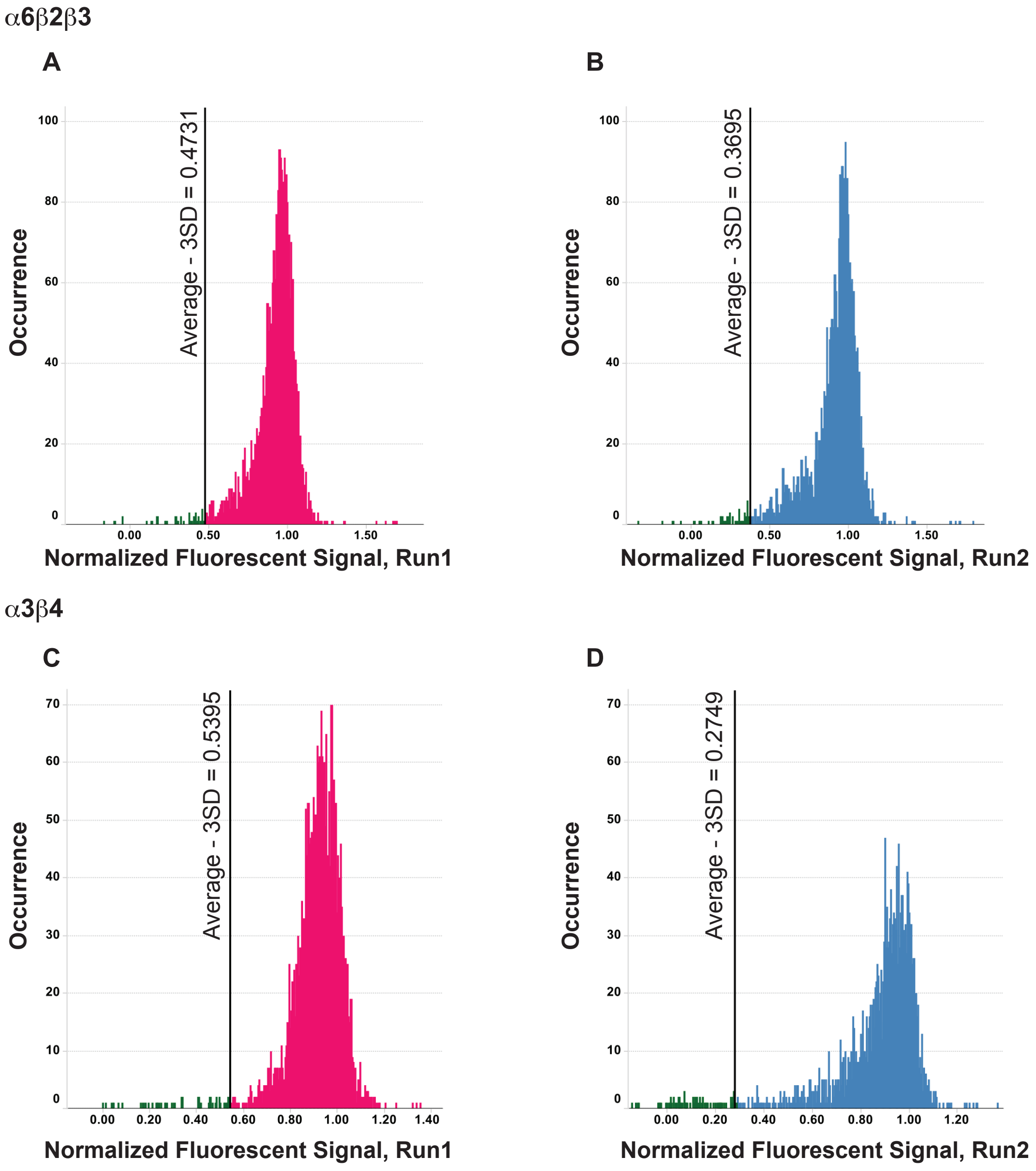

Supplemental Figure 3. Hit selection for SH-EP1-α3β4-nAChR and SH-EP1-α6β2β3-nAChR.
